# Differences in the neural correlates of schizophrenia with positive and negative formal thought disorder in patients with schizophrenia in the ENIGMA dataset

**DOI:** 10.1038/s41380-024-02563-z

**Published:** 2024-04-26

**Authors:** Rachel J. Sharkey, Chelsea Bacon, Zeru Peterson, Kelly Rootes-Murdy, Raymond Salvador, Edith Pomarol-Clotet, Andriana Karuk, Philipp Homan, Ellen Ji, Wolfgang Omlor, Stephanie Homan, Foivos Georgiadis, Stefan Kaiser, Matthias Kirschner, Stefan Ehrlich, Udo Dannlowski, Dominik Grotegerd, Janik Goltermann, Susanne Meinert, Tilo Kircher, Frederike Stein, Katharina Brosch, Axel Krug, Igor Nenadic, Kang Sim, Gianfranco Spalletta, Nerisa Banaj, Scott R. Sponheim, Caroline Demro, Ian S. Ramsay, Margaret King, Yann Quidé, Melissa Jane Green, Dana Nguyen, Adrian Preda, Vince Calhoun, Jessica Turner, Theo van Erp, Thomas Nickl-Jockschat

**Affiliations:** 1 Iowa Neuroscience Institute, Carver College of Medicine, University of Iowa, Iowa City, IA USA; 2 Department of Psychiatry, University of Iowa, Iowa City, IA USA; 3 Department of Psychology,, Georgia State University, Atlanta, GA USA; 4 FIDMAG Germanes Hospitalàries Research Foundation, CIBERSAM ISCIII, Barcelona, Spain; 5 Department of Psychiatry, Psychotherapy and Psychosomatics, Psychiatric University Hospital Zurich (PUK), Zurich, 8008 Switzerland; 6 Department of Psychiatry, Geneva University Hospitals, Geneva, Switzerland; 7 Translational Developmental Neuroscience Section, Division of Psychological and Social Medicine and Developmental Neurosciences, Faculty of Medicine, TU Dresden, Dresden, Germany; 8 Institute for Translational Psychiatry, University of Münster, Münster, Germany; 9 Department of Psychiatry and Psychotherapy, University of Marburg, Marburg, Germany; 10 Department of Psychiatry and Psychotherapy, University of Bonn, Bonn, Germany; 11 West Region, Institute of Mental Health, Singapore, Singapore; 12 Laboratory of Neuropsychiatry, IRCCS Santa Lucia Foundation, Rome, Italy; 13 Department of Psychiatry and Behavioral Sciences, University of Minnesota, Minneapolis, MN USA; 14 The MIND Research Network, Albuquerque, NM USA; 15 School of Psychiatry, University of New South Wales (UNSW) Sydney, Sydney, NSW Australia; 16 Department of Pediatric Neurology, University of California Irvine, Irvine, CA USA; 17 Department of Psychiatry and Human Behavior, University of California Irvine, Irvine, USA; 18 Tri-institutional Center for Translational Research in Neuroimaging and Data Science (TReNDS), Georgia State University, Georgia Institute of Technology, Emory University, Atlanta, GE USA; 19 Department of Psychiatry and Behavioral Medicine, Ohio State University, Columbus, OH USA; 20 Center for the Neurobiology of Learning and Memory, University of California Irvine, Irvine, CA USA; 21 Clinical Translational Neuroscience Laboratory, Department of Psychiatry and Human Behavior, University of California Irvine, Irvine, USA; 22 Department of Neuroscience and Pharmacology, Carver College of Medicine, University of Iowa, Iowa City, IA USA; 23 Department of Psychiatry and Psychotherapy, Otto-von-Guericke University, Magdeburg, Germany; 24 German Center for Mental Health (DZPG), partner site Halle-Jena-Magdeburg, Magdeburg, Germany; 25 Center for Intervention and Research on adaptive and maladaptive brain Circuits underlying mental health (C-I-R-C), Halle-Jena-Magdeburg, Magdeburg, Germany

**Keywords:** Schizophrenia, Neuroscience

## Abstract

Formal thought disorder (FTD) is a clinical key factor in schizophrenia, but the neurobiological underpinnings remain unclear. In particular, the relationship between FTD symptom dimensions and patterns of regional brain volume loss in schizophrenia remains to be established in large cohorts. Even less is known about the cellular basis of FTD. Our study addresses these major obstacles by enrolling a large multi-site cohort acquired by the ENIGMA Schizophrenia Working Group (752 schizophrenia patients and 1256 controls), to unravel the neuroanatomy of FTD in schizophrenia and using virtual histology tools on implicated brain regions to investigate the cellular basis. Based on the findings of previous clinical and neuroimaging studies, we decided to separately explore positive, negative and total formal thought disorder. We used virtual histology tools to relate brain structural changes associated with FTD to cellular distributions in cortical regions. We identified distinct neural networks positive and negative FTD. Both networks encompassed fronto-occipito-amygdalar brain regions, but positive and negative FTD demonstrated a dissociation: negative FTD showed a relative sparing of orbitofrontal cortical thickness, while positive FTD also affected lateral temporal cortices. Virtual histology identified distinct transcriptomic fingerprints associated for both symptom dimensions. Negative FTD was linked to neuronal and astrocyte fingerprints, while positive FTD also showed associations with microglial cell types. These results provide an important step towards linking FTD to brain structural changes and their cellular underpinnings, providing an avenue for a better mechanistic understanding of this syndrome.

## Introduction

Formal thought disorder (FTD) is a syndrome characterized by disorganized and incoherent speech [1, 2]. FTD is a cross-diagnostic syndrome, but it constitutes a key clinical factor of schizophrenia and a component of the diagnostic criteria in the DSM-5. Multiple lines of evidence point towards a key role for FTD in the pathophysiology of schizophrenia. It predicts transition into psychosis in clinical high-risk samples [3–5] and is closely correlated with long-term outcome in chronified disease states [6–9].

FTD is a clinically highly heterogeneous syndrome, with impairments ranging from impoverished thought to disorganized thinking to pressured speech [10]. These different facets differentially impact clinical outcomes [7, 9, 11]. To reduce this heterogeneity, researchers have suggested grouping FTD in positive and negative symptoms [12–14]. Similarly to the overall positive and negative symptom dimensions of schizophrenia, positive formal thought disorder symptoms are characterized by disorganized or unusual forms of thought or language, while negative formal thought disorder symptoms include paucity or slowing of thought or speech [10].

Brain volume loss in fronto-temporo-basal ganglia-thalamic networks is a hallmark of schizophrenia pathology [15, 16] and might provide a major avenue towards unraveling the neurobiological basis of FTD. These brain structural changes are progressive over the disease course [15] and, at least retrospectively, linked to symptom patterns [17, 18]. Although there is an established relationship between volume loss and overall symptom patterns in schizophrenia, major questions remain, specifically about the neuroanatomical basis of FTD in schizophrenia [16, 17, 19]. Several pioneering publications reported brain structural correlates of FTD, but these studies enrolled comparatively small numbers of schizophrenia patients (20-30 patients) [20–22]. Studies on much larger samples of thousands of patients, on the other hand, pool across patients with various diagnoses [23–25]. This approach reduces the risk of spurious correlations and increases the power compared to smaller samples; it also has the advantage that it can identify transdiagnostic markers of FTD. However, it might be less sensitive towards pathologies associated specifically with schizophrenia, as their progressive nature, their location and extent might set them apart from brain structural changes observed in other disorders [15, 26, 27].

Finally, a major limitation of conventional structural neuroimaging is that its findings can reflect various histological correlates and are, necessarily, neurobiologically nonspecific. Quantitative neuroimaging techniques (cf. ref. [28]), which can provide a greater degree of physiological specificity, but these require specialized MR sequences and there are, to our knowledge, no large multi-center samples of quantitative MR patients with schizophrenia Prior work in *post-mortem* samples has suggested that multiple neuronal and glial cell types are likely involved in the pathophysiology of schizophrenia, making histological specificity especially important [29–32]. This lack of specificity in structural neuroimaging is a major obstacle when it comes to the identification of cellular mechanisms and neuronal circuits associated with FTD, which is difficult to model in animals. However, while neuroimaging even at ultra-high magnet strengths cannot provide a sufficient resolution to capture this level of detail, modern computational approaches based on gene expression data allow inference on the cellular composition of a given brain region [33–35]. These approaches have been subsumed under the umbrella term “virtual histology”. While this technique still relies on a relatively small pool of data, the difficulty of accessing human gene expression within the brain makes this a powerful and invaluable technique.

Our study addresses these major obstacles to unravel the neuroanatomical basis of formal thought disorder by enrolling a large multi-site cohort and using virtual histology tools on implicated brain regions. In detail, we present the first study using the large, multi-site ENIGMA dataset to identify structural correlates of FTD. As previous neuroimaging studies [36, 37] have hinted at least at distinct functional correlates of positive and negative FTD, we decided to separately explore different symptom dimensions, namely positive, negative and total formal thought disorder. We secondly used virtual histology tools to relate the study’s structural findings to cellular distributions in the cortex and identify possible contributing factors at the cellular level, to our neuroimaging findings. To our knowledge, this study enrolls the largest existing schizophrenia cohort to study structural correlates of FTD, and the first to apply a virtual histology approach to these findings.

## Results

### Structural associations with formal thought disorder

We constructed a general linear model associating formal thought disorder scores with regional surface areas or cortical thickness measurements of each brain region, controlling for age, age squared, gender and intracranial volume. Separate models were used for total (PANSS items: P2, N5, N6, N7), positive (P2) and negative (N5, N6, N7) formal thought disorder, and, finally, for each of the three PANSS items contributing to negative formal thought disorder separately. This definition of formal thought disorder has been used previously in investigating the neural basis of formal thought disorder [34].

### Total formal thought disorder

**Fig. 1:**
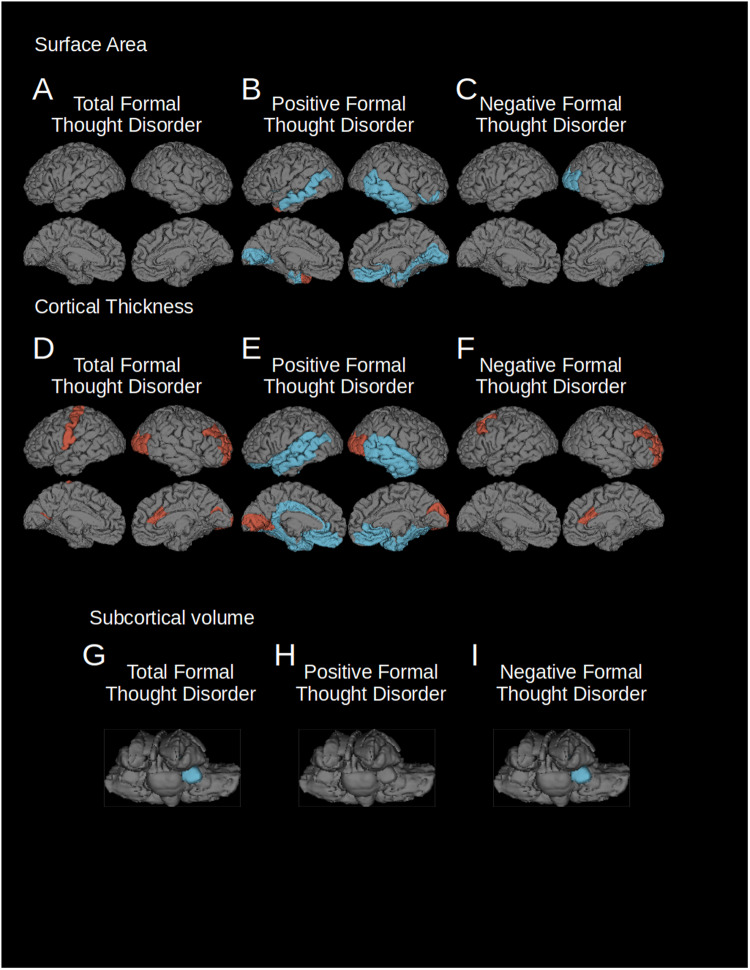
Regions where cortical surface area or thickness were significantly associated with total, positive and negative FTD (FDR corrected *p* = 0.001). Left hemisphere is shown on the left of each group of images. Blue indicates regions of reduced surface area, cortical thickness or subcortical volumes, red indicates regions of relatively spared surface area, cortical thickness or subcortical volumes. **A** Surface area changes associated with total FTD; **B** surface area changes associated with positive FTD; **C** surface area changes associated with negative FTD; **D** cortical thickness changes associated with total FTD; **E** cortical thickness changes associated with positive FTD; **F** cortical thickness changes associated with negative FTD; **G** volume changes of subcortical structures associated with total FTD; **H** volume changes of subcortical structures associated with positive FTD; **I** volume changes of subcortical structures associated with negative FTD.

Total formal thought disorder was associated with reductions in the volumes of the bilateral pallidum. It was associated with increased cortical thickness in fronto-occipital regions. Increased cortical thickness in rostral middle frontal and postcentral pole regions, as well as the right caudal anterior cingulate cortex and lateral occipital and pericalcarine regions were implicated (Fig. 1A, D, G; Table 1).

**Table 1:** T statistics of regions significantly associated with total FTD (FDR corrected *p* = 0.001).

Surface area	Cortical thickness				Volume			
			Right Lateral Occipital	4.79	Left Pallidum	−5.92	Right Pallidum	−5.10
	Left Pericalcarine	5.08	Right Pericalcarine	4.78				
	Left Post Central	4.58						
			Right Rostral Middle Frontal	5.15				
			Right Caudal Anterior Cingulate	5.03				

### Positive formal thought disorder

In our study, positive formal thought disorder was associated with neuroanatomical changes in frontal, temporal and occipital cortical regions. We found reductions in surface area in the bilateral temporal, bilateral lingual, and right medial orbitofrontal cortex. It was also associated with reductions in cortical thickness in the left lateral orbitofrontal and bilateral medial orbitofrontal cortex, the rostral anterior cingulate, left caudal anterior and posterior cingulate and temporal cortex (including the superior and middle temporal gyri and temporal poles) the parahippocampal, entorhinal and fusiform cortex, and the insula. There were regions of increased cortical thickness in the right cuneus, pericalcarine and lateral occipital cortex and left lingual and, pericalcarine (Fig. 1B, E, H; Table 2).

**Table 2:** T statistics of regions significantly associated with positive FTD (*p* = 0.001, FDR corrected).

Surface area				Cortical thickness				Volume
						Right Lateral Occipital	4.36	
						Right Cuneus	4.34	
Left Lingual	−4.29	Right Lingual	−5.59	Left Lingual	4.78			
Left Pericalcarine	−4.90	Right Pericalcarine	−5.75	Left Pericalcarine	7.32	Right Pericalcarine	7.27	
		Right Medial Orbitofrontal	−4.81	Left Medial Orbitofrontal	−4.12	Right Medial Orbitofrontal	−4.43	
		Right Inferior Temporal	−4.84	Left Lateral Orbitofrontal	−5.61			
Left Middle Temporal	−4.10	Right Middle Temporal	−4.42	Left Rostral Anterior Cingulate	−7.62	Right Rostral Anterior Cingulate	−6.90	
Left Temporal Pole	4.41							
		Right Parahippocampal	−4.33	Left Caudal Anterior Cingulate	−4.58			
Left Insula	−4.30	Right Insula	−5.97	Left Posterior Cingulate	−5.12			
				Left Isthmus Cingulate	−5.66			
				Left Temporal Pole	−5.75	Right Temporal Pole	−6.47	
				Left Banks of Superior Temporal Sulcus	−6.95	Right Banks of Superior Temporal Sulcus	−6.48	
				Left Superior Temporal	−5.43	Right Superior Temporal	−6.18	
				Left Middle Temporal	−4.61	Right Middle Temporal	−7.90	
						Right Inferior Temporal	−5.78	
				Left Fusiform	−6.55	Right Fusiform	−5.90	
Left Entorhinal	−4.21	Right Entorhinal	−4.29	Left Entorhinal	−6.08	Right Entorhinal	−5.53	
				Left Parahippocampal	−4.89	Right Parahippocampal	−4.68	
				Left Insula	−6.14	Right Insula	−4.88	
		Right Pars Orbitalis	−4.10					

### Negative formal thought disorder

We identified neuroanatomical changes mainly in frontal, occipital and subcortical regions associated with negative formal thought disorder. Surface areas were reduced in the lateral occipital. Reduced volume in the pallidum was also associated with this symptom dimension. Increased cortical thickness in the right caudal anterior cingulate right rostral middle frontal gyri, and the left caudal middle frontal lobe were correlated with negative formal thought disorder. When the three PANSS scores contributing to the negative formal thought disorder score were examined separately, the significant findings were predominantly driven by associations with N5; difficulty with abstract thinking. N6 was not significantly associated with cortical volume and N7 was associated only with increased medial orbitofrontal thickness (Fig. 1C, F, I; Table 3; Supplementary Table 3).

**Table 3:** T statistics of regions significantly associated with negative FTD (*p* = 0.001, FDR corrected).

Surface area		Cortical thickness				Volume			
Right Lateral Occipital	−4.57			Right Rostral Middle Frontal	5.48	Left Pallidum	−5.73	Right Pallidum	−5.30
				Right Caudal Anterior Cingulate	5.43				
		Left Caudal Middle Frontal	4.59						

While a fronto-occipital network was implicated in all three symptom dimensions–total, positive and negative FTD–frontal changes, in particular those in the medial orbitofrontal cortex, showed opposite patterns of cortical thickness: positive FTD was associated with *decreased* cortical thickness in that brain region, while total and negative FTD were correlated with *increased* cortical thickness. Lateral temporal regions were implicated only in positive FTD. These findings suggest a neurobiological divergence of positive and negative FTD and are in line with recent functional neuroimaging findings [19, 36].

### The influence of site on the results

**Fig. 2:**
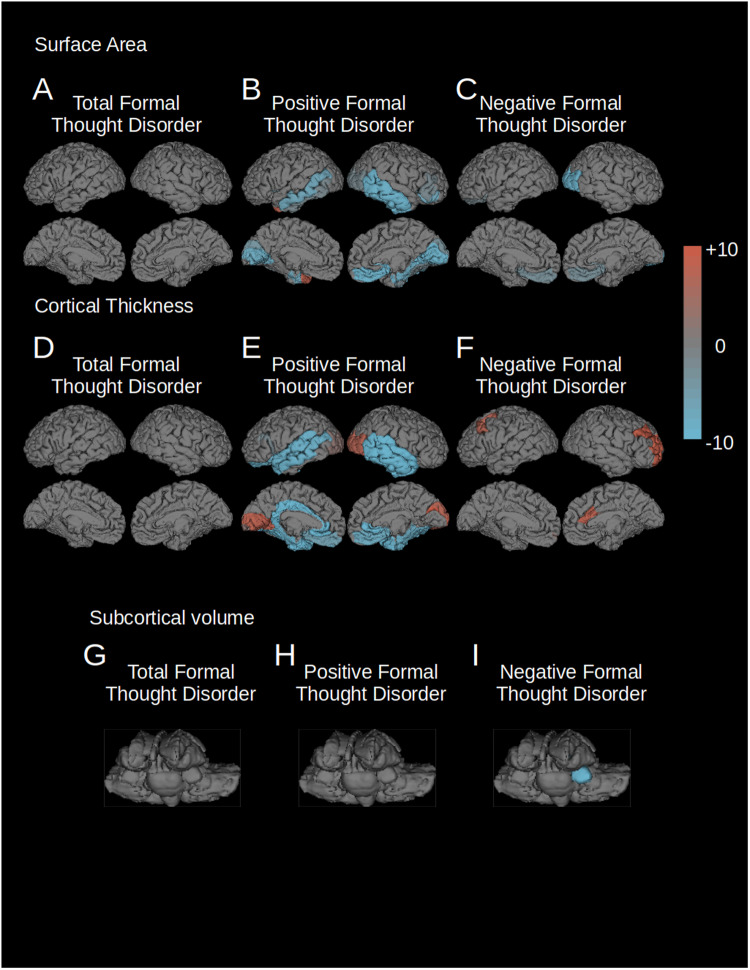
“Leave one out” analysis of FTD correlation. The regions associated with total, positive, or negative FTD (FDR corrected *p* = 0.001) of each of the 10 “leave one out” analyses were added together so that all positively affected regions are shown in red, and all negatively affected regions in blue. The intensity of the color represents the number of “leave one out” analyses where that region was affected. Leave one out analyses were conducted for the following parameters: **A** surface area changes associated with total FTD; **B** surface area changes associated with positive FTD; **C** surface area changes associated with negative FTD; **D** cortical thickness changes associated with total FTD; **E** cortical thickness changes associated with positive FTD; **F** cortical thickness changes associated with negative FTD; **G** volume changes of subcortical structures associated with total FTD; **H** volume changes of subcortical structures associated with positive FTD; **I** volume changes of subcortical structures associated with negative FTD.

To control for site-specific effects on our results, we conducted a leave-one-out analysis. Results were consistent for positive and negative FTD, while findings for total FTD did not reproduce (Fig. 2, Tables 4 and 5). We interpreted these results as indicative of robust findings for both FTD subscales, while structural correlates of total FTD seemed to be influenced by site effects.

**Table 4:** T statistics of regions significantly associated with positive FTD after “leave one out” analysis.

Surface area				Cortical thickness				Volume
		Right Lateral Occipital*	−2	Left Lateral Occipital*	2	Right Lateral Occipital*	7	
Left Cuneus	−4	Right Cuneus	−3			Right Cuneus*	7	
Left Lingual*	−8	Right Lingual*	−9	Left Lingual*	8			
Left Pericalcarine*	−9	Right Pericalcarine*	−10	Left Pericalcarine*	10	Right Pericalcarine*	10	
		Right Medial Orbitofrontal*	−9	Left Medial Orbitofrontal	−6	Right Medial Orbitofrontal*	−8	
Left Inferior Temporal*	−2	Right Inferior Temporal*	−9	Left Lateral Orbitofrontal*	−9			
Left Middle Temporal	−7	Right Middle Temporal*	−9	Left Rostral Anterior Cingulate*	−10	Right Rostral Anterior Cingulate*	−10	
Left Temporal Pole*	8							
		Right Parahippocampal*	−8	Left Caudal Anterior Cingulate*	−9			
Left Insula*	−9	Right Insula*	−9	Left Posterior Cingulate*	−10			
				Left Isthmus Cingulate*	−10			
				Left Temporal Pole*	−10	Right Temporal Pole*	-10	
		Right Banks of Superior Temporal Sulcus	−2	Left Banks of Superior Temporal Sulcus*	−10	Right Banks of Superior Temporal Sulcus*	−10	
				Left Superior Temporal*	−9	Right Superior Temporal*	−10	
				Left Middle Temporal*	−9	Right Middle Temporal*	−10	
						Right Inferior Temporal*	−10	
		Right Fusiform	−2	Left Fusiform*	−10	Right Fusiform*	−9	
Left Entorhinal*	−7	Right Entorhinal*	−8	Left Entorhinal*	−10	Right Entorhinal*	−9	
				Left Parahippocampal*	-9	Right Parahippocampal*	-8	
				Left Insula*	−10	Right Insula*	−9	
		Right Pars Orbitalis	−7					
		Right Pars Triangularis	−2					
				Left Pars Opercularis	−2			

Results of the leave-one-out (LOO) analyses for positive FTD. All regions are displayed that were significant for at least one LOO analysis (*p* = 0.001, FDR corrected). Numbers provided indicate the number of times that these regions were retrieved as significant during the individual LOO runs. Positive numbers indicate positive correlations with FTD, negative numbers indicate negative correlations. Regions that were retrieved by the main analysis are displayed in bold. The value displayed is the summary of the direction of significant t-tests in each region, ranging from [−10,10] with −10 meaning the region showed negative correlation in all 10 LOO analyses, and 10 meaning the region showed positive correlation in all 10 LOO analyses. Regions that were also significant in the main analysis are displayed with an asterisk *.

**Table 5:** T statistics of regions significantly associated with negative FTD after “leave one out” analysis.

Surface area				Cortical thickness				Volume			
		Right Lateral Occipital*	−9					Left Pallidum*	−9	Right Pallidum*	−9
						Right Rostral Middle Frontal*	9				
						Right Caudal Anterior Cingulate*	8				
Left Lateral Orbitofrontal	−2										
		Right Cuneus	−2								
Left Medial Orbitofrontal	−3	Right Medial Orbitofrontal	−3	Left Medial Orbitofrontal	2	Right Medial Orbitofrontal	2				
				Left Frontal Pole	2	Right Frontal Pole	2				
		Right Insula	−2								
				Left Caudal Middle Frontal	7						

Results of the leave-one-out (LOO) analyses for negative FTD. All regions are displayed that were significant for at least one LOO analysis (*p* = 0.001, FDR corrected). Numbers provided indicate the number of times that these regions were retrieved as significant during the individual LOO runs. Positive numbers indicate positive correlations with FTD, negative numbers indicate negative correlations. Regions that were retrieved by the main analysis are displayed in bold. The value displayed is the summary of the direction of significant t-tests in each region, ranging from [−10,10] with −10 meaning the region showed negative correlation in all 10 LOO analyses, and 10 meaning the region showed positive correlation in all 10 LOO analyses. Regions that were also significant in the main analysis are displayed with an asterisk *.

### Patient vs. control comparisons

In order to compare results related to formal thought disorder with those more generally associated with schizophrenia, we used a two-sample t-test comparing patients with schizophrenia with controls. We identified wide-spread reductions in cortical surface area and volume, reduced subcortical volume, and increased lateral ventricle volume in patients compared to healthy controls, but also increased left caudate volume in the patients. The regions of relative increase compared to other patients identified in the analysis of FTD were still reduced compared to the control group, indicating a process of relative sparing rather than genuine increase (Supplementary Table 2).

### Auditory hallucination associations

Prior research has led to the hypothesis that FTD primarily emerges from disturbances in language processing networks (dyssemantic hypothesis of FTD) [38]. To test whether the identified structural changes were specific for FTD or rather indicative of unspecific changes in language processing networks in schizophrenia [2], we explored potential correlations between structural variation in FTD-related brain regions and auditory hallucinations, another major language-associated pathology in schizophrenia [39]. The regions which were significantly associated with FTD were also examined for a relationship with auditory hallucination symptoms using similar general models. This resulted in no significant findings, suggesting this network is specific to formal thought disorder, not general to auditory and language dysfunction in schizophrenia.

### Cellular genetic fingerprint associations

MRI imaging has provided valuable insight into in vivo pathologies in schizophrenia, but is unable to provide information about underlying histological changes. Novel virtual histology approaches based on gene expression databases utilize complex gene expression patterns to identify the cellular composition of a given brain region [33, 34]. Capitalizing on this approach, we correlated the distribution of these gene expression fingerprints with the patterns of cortical thickness change identified in our data as described previously [33]. In brief, he Freesurfer processing pipeline was run as described in the ENIGMA Consortium documents, generating cortical thickness/thinning values for regions defined by the Desikan-Killiany Atlas. We, then, relied upon the Allen Human Brain Atlas [40], which provides unique resource, as it contains expression levels of >62,000 genes and isoforms from 3702 samples (punch biopsies) taken from 6 different donor brains. The exact locations of these samples were mapped to MNI space. This allows a joint analysis of transcriptomic data with neuroimaging findings. Transcriptomic markers for 9 neural cell types (S1 pyramidal, CA1 pyramidal, interneuron, astrocyte, microglia, oligodendrocyte, ependymal, endothelial, and mural) were, then, correlated with the regional thickness/thinning values in order to extrapolate cell type specific contributions to effect. All FTD dimensions were bilaterally associated with transcriptomic fingerprints for astrocytes (“astrocyte”) and dendritic spine maintenance (“CA1 pyramidal”). The “CA1 pyramidal” label here refers to the original source for the genetic fingerprint, but the respective gene expression patterns have been confirmed to be distributed through the cortex [33]. Positive FTD was also associated with the fingerprint for microglial function (“microglia”), but negative formal thought disorder was associated with microglial function in the right hemisphere. Hence, the brain structural dissociation between positive and negative FTD appears to be somewhat accompanied by a dissociation also on a cellular level (Table 6). Positive FTD, which was associated with greater cortical atrophy was also more strongly associated with microglia which have been previously associated with excessive synaptic pruning in schizophrenia [41].

**Table 6:** FDR corrected *P*-values for associations between FTD related cortical thickness changes and distributions of cellular transcriptomic signatures.

		Astrocyte	Dendritic maintenance (CA1 pyramidal)	Microglia
Total formal thought disorder	Left	0.0013	0.00090	0.048
	Right	0.00045	0.00045	0.0060
Positive formal thought disorder	Left	0.00090	0.00090	0.037
	Right	0.0067	0.00090	0.0069
Negative formal thought disorder	Left	0.0081	0.00090	
	Right	0.011	0.0018	0.013

## Discussion

This study identified novel neural networks associated with different symptom dimensions of formal thought disorder in a large cohort of schizophrenia patients (Fig. 1). While prior studies of formal thought disorder, including meta-analyses have identified changes in superior temporal gyrus activation [42] and connectivity associated with formal thought disorder–the latter specifically for positive formal thought disorder [35] – this study suggests that these earlier findings were only one component of the more extensive networks we have identified. The temporal component of the structural network identified in this study overlaps with previously identified functional findings but connects to a wider range of areas.

Both positive and negative FTD were found to impact fronto-occipito brain regions, namely the medial orbitofrontal cortex, anterior cingulate, lateral occipital cortex and negative FTD was also found to impact the left amygdala. However, anatomical measures were impacted differentially across the two FTD dimensions. The functional associations of our FTD core network provide further insight into a long-standing controversy in the field: whether FTD emerges from dysfunction in language processing networks (“dyssemantic hypothesis”) [43] or rather from deficits in higher-order cognitive processes (“dysexecutive hypothesis”) [38]. Of note, these core regions were outside canonical language-related circuits [44], but rather associated with cognitive and behavioral control (*medial orbitofrontal* [45–47] and *anterior cingulate* [48, 49]), affective processing (*amygdala* [50, 51]) which have been associated with schizophrenia in previous studies [16, 20, 52], and abstract thinking and imagination (*lateral occipital cortex* [53–57]), which has been less commonly associated with schizophrenia [56, 58, 59]. Our findings suggest a potential role for dysfunctional executive processing as a common feature shared across FTD domains. The orbitofrontal cortex and amygdala have also been associated with top-down visual processing abnormalities in schizophrenia. However, it should be noted that positive FTD was associated also with classical language-related regions, suggesting a role for impaired semantic functions especially in the case of positive FTD (Fig. 1B, E; Tables 1–3) [24, 60].

A closer look on differences between findings for distinct FTD symptom dimensions demonstrates a dissociation between the structural features of positive and negative formal thought disorder. In particular, positive and negative FTD showed opposing patterns of associations with cortical thickness in the orbitofrontal cortex and the rostral anterior cingulate (Fig. 1B, C, E, F). Negative FTD showed a positive correlation with cortical thickness in these frontal brain regions, however, it should be noted that these findings were indicative of a relative sparing from a fronto-temporal pattern of atrophy [16, 20] in our patient sample, rather than an absolute increase, when compared to healthy controls (Fig. 1). When our sample was compared to healthy controls, it showed a pattern of widespread reductions in cortical surface area and thickness and subcortical volume (Supplementary Table 2). Different atrophy patterns in these two central hubs involved in cognitive control could indicate differential biological mechanisms or different cellular populations that play a role in the emergence of formal thought disorder. Additionally, positive FTD was the only symptom dimension that implicated brain regions in the temporal cortex, particularly in language-related parts (Fig. 1B, E; Table 2). A previous meta-analysis from our lab has highlighted functional changes of the superior and medial temporal gyrus in FTD, central hubs of the human language processing network [59, 61]. The temporal pole, in turn, has been linked to a semantic network involved in creative thinking [62]. Importantly, connectome-based modeling with seeds in the superior and medial temporal cortex allowed a prediction of individual symptom severity for positive FTD, but not any other FTD dimension [19], which is well in line with our own findings. Together, these findings paint the picture of a role for language-related networks exclusively in positive formal thought disorder. The results of our study further support the idea of a fundamental neurobiological divergence between positive and negative symptom dimensions [1, 63] that has been shown for general schizophrenia psychopathology and neural correlates respectively [19]. Existing imaging research on FTD has also identified multiple networks using different MR modalities [24, 60]. While these networks are not identical to the ones identified in this study, they do show some compelling overlaps. Frontotemporal networks associated with FTD have been identified in both groups of patients with schizophrenia-spectrum disorders only, and in cross-diagnostic patient populations. Interestingly, a very large study of patients on the schizophrenia spectrum, identified increased occipital fractional anisotropy was negatively associated with one aspect of FTD (disorganized), but occipital findings were not identified in a smaller study of patients with schizophrenia or with a transdiagnostic sample.

Due to its limited spatial resolution, MR imaging does not allow a direct link between macroscopic changes and underlying molecular or cellular pathologies, a key requirement for the development of new therapeutic approaches. Novel methods, however, allow at least indirect inference on these molecular processes. Our virtual histology approach [33], based on gene expression patterns provided by the Allen Human Brain Atlas [40], identified distinct transcriptomic fingerprints associated with each of the three symptom dimensions (Table 6). Common to both the positive and negative FTD dimensions was a transcriptomic signature associated with dendritic spine maintenance and astrocytes, which were also the only fingerprint found for brain regions associated with negative FTD. Reduced dendritic spine density and impaired dendritic plasticity has been frequently reported in brains of schizophrenia patients *post mortem* [32, 64]. Mechanistically, loss of dendritic spines has been linked to altered function in human complex 4 (C4) [65]. Complex genetic variation in the C4 gene, in turn, has been linked to schizophrenia risk [66]. Our own finding that neuroanatomical variation associated with both FTD dimensions is situated in brain regions with a high demand for dendritic spine maintenance appears as plausible in light of these previous findings. Besides their role in synapse formation during development [67], astrocytes are known to modulate glutamatergic signaling [28, 30]. Pharmacological antagonization of glutamate signaling, in turn, has been shown to induce both positive and negative FTD in healthy subjects [68–70]. Beyond these signatures, positive formal thought disorder was also found to be associated with brain regions enriched for another non-neuronal cellular fingerprint: microglia. As resident immune cells of the central nervous system, microglia cells are involved in synaptic pruning during development [41, 71].

Our study has several limitations. The extremely large sample size of our study made possible by the ENIGMA consortium has enabled us to identify novel neural networks associated with FTD that earlier, smaller studies were unable to identify. This advantage, however, comes at the cost of rather unspecific rating scales for FTD. Future prospective studies might add additional insights by using rating scales specifically designed for FTD, such as the TLC [72] or the TALD [73]. The PANSS assigns scores between 0–7 for individual items, which leads to a comparatively coarse assessment of distinct symptom dimensions. In addition, FTD symptoms in schizophrenia occur in the context of the other symptoms of the disorder. An exploratory analysis of the impact of the remaining symptom scores on the cortical thickness findings revealed a greater degree of sparing of cortical thickness and volume in the previously described frontotemporal-occipital, and fewer regions exhibiting cortical thinning survived the multiple comparisons correction, while the results showed the same overall pattern (Supplementary Table 4). To fully explore this impact a more tailored sample containing more participants where the two are less correlated should be employed. Additionally, symptom severity is inherently limited by the ability of patients to give informed consent. Further, neuroanatomical changes in schizophrenia have been shown to be progressive. Hence, a longitudinal approach might provide an even better link between brain structural alterations and FTD than our cross-sectional approach and will be an important aim for future studies. This approach also limited the level of detail we could examine these associations with. There are several other aspects of the disease process which are known to impact cortical volume in schizophrenia which could impact these associations, including medication effects and overall cognitive status.Finally, we used a recently established virtual histology approach to identify potential cellular contributions to FTD [33]. Finally, a major limitation of conventional structural neuroimaging is that its findings can reflect various histological correlates and are, necessarily, neurobiologically nonspecific. Quantitative neuroimaging techniques (cf. ref. [28]), which can provide a greater degree of physiological specificity, but these require specialized MR sequences and there are, to our knowledge, no large multi-center samples of quantitative MR patients with schizophrenia.

In sum, this study demonstrates a convergence between neuroimaging and cellular endophenotypes and is, to the best of our knowledge, the first to associate glial function with formal thought disorder specifically. The identification of a multi-scale associations between structural and transcriptomic networks associated with cellular function is of specific interest clinically, because it provides the basis for linking neuroimaging findings and clinically relevant molecular targets in a way that is not possible with either method in isolation.

## Methods

### ENIGMA data

This study used the data from 752 patients and 1256 controls from the ENIGMA Consortium Schizophrenia Working Group. The data used included cortical thickness, cortical surface area, and subcortical volume for each region in the Desikan-Killiany atlas,as well as demographic information and PANSS symptom scores. We used the established quality control pipelines for MRI data sets of the ENIGMA consortium (https://enigma.ini.usc.edu/protocols/imaging-protocols/). Refer to ENIGMA Consortium documents for more details on subject recruitment and data collection and processing (Supplementary Table 1).

### Formal thought disorder scores

To measure the degree of the four formal thought disorder symptoms being assessed for each patient we calculated a symptom score based on a subset of symptom subscales from the PANSS. Four symptom scores were calculated for each patient: total formal thought disorder, positive formal thought disorder, negative formal thought disorder and hallucinations.

The total formal thought disorder scale was derived from the sum of items P2, N5, N6 and N7, as has been used in previous studies [36]. Of this subset, P2 was used as a measure of positive formal thought disorder, while negative formal thought disorder was measured as the sum of N5, N6 and N7. Hallucinations were measured by the P3 score. Tests of the relationship between formal thought disorder related findings and auditory hallucinations were carried out to test the specificity of the results to formal thought disorder.

### General linear models

Each of the major comparisons in this study used a general linear model examining associations between a variable of interest and the regional cortical thickness, surface area and subcortical volume values in a mass univariate manner which was then controlled for multiple comparisons to false discovery rate q = 0.001. As previous research strongly suggests a clinical and neurobiological positive-negative dichotomy not only for schizophrenia psychopathologz, in general, but for FTD symptoms, in specific, we chose to conduct separate analyses for positive, negative and total FTD. Each model controlled for age, age squared, gender and intracranial volume in format:

$${{{{{\rm{Y}}}}}}= 	 \, {{{{{{\rm{\beta }}}}}}}_{0}+{{{{{{\rm{\beta }}}}}}}_{1}\{{{{{{\rm{Variable}}}}}}\; {{{{{\rm{of}}}}}}\; {{{{{\rm{interest}}}}}}\}+{{{{{{\rm{\beta }}}}}}}_{2}{{{{{\rm{Age}}}}}}+{{{{{{\rm{\beta }}}}}}}_{3}{{{{{{\rm{Age}}}}}}}^{2}\\ 	 + \, {{{{{{\rm{\beta }}}}}}}_{4}{{{{{\rm{Gender}}}}}}+{{{{{{\rm{\beta }}}}}}}_{5}{{{{{\rm{Intracranial\; Volume}}}}}}+{{{{{\rm{\varepsilon }}}}}}$$

This model was used for the relationship between cortical thickness and surface area and subcortical volume and: Total Formal Thought Disorder, Positive Formal Thought Disorder, Negative Formal Thought Disorder, N5, N6, N7 and Diagnosis. The same model was applied to the relationship between Auditory Hallucinations and regions significantly correlated with the formal thought disorder scores. Given that whole brain atrophy has been consistently described in schizophrenia, we have opted for TIV as most suitable covariate.Models of this sort have previously been used in multiple ENIGMA studies [74, 75].

### Leave one out data validation for site variability

Matlab scripts were written to check for any site-specific variability in the data. This analysis was performed by sequentially removing data from each site one at a time and performing the statistics on the reduced dataset. We then created a summary statistic from the regions with significant associations (*p* < 0.001) by summing the direction of the t-test, i.e. add 1 for positive significant association, and subtract 1 for negative significant association, giving a possible range of [−10,10], depending on the number of repeated associations.

### Patient vs. control t-test

A bidirectional two-sample t-test was performed comparing the patient and control groups, without controlling for the additional variables of no interest. This was included along with the GLM for diagnosis effects to model effects which include the global reduction in ICV which has been previously demonstrated in schizophrenia.

### Cellular genetic fingerprint associations

R scripts released by the Paus Lab were used to associate our regional cortical thickness findings with the regional distributions of different cellular fingerprints. Refer to Shin et al. for the complete methods [33].

## Supplementary information

Supplemental Figures and Tables Captions

Supplemental Table 1

Supplemental Table 2

Supplemental Table 3

Supplemental Figure 1

Supplemental Figure 2

Supplemental Figure 3

Supplemental Table 4

## Data Availability

The R code used to perform the virtual histology analyses are stored at the Paus lab.
