# Supplementary material for: Differences in the neural correlates of schizophrenia with positive and negative formal thought disorder in patients with schizophrenia in the ENIGMA dataset
Source: Mol Psychiatry. 2024 Apr 26;29(10):3086–96. doi: 10.1038/s41380-024-02563-z (PMC11449795; doi:10.1038/s41380-024-02563-z)
Supplement: Supplementary file 1 — Supplemental Figures and Tables Captions [file 41380_2024_2563_MOESM1_ESM.docx]

**Supplement**

**Captions for Supplemental Figures and Tables**

**Supplemental Figure 1**

Boxplot PANSS item scores of schizophrenia patients included in this study.

**Supplemental Figure 2**

Boxplot of PANSS item scores used to determine FTD symptom levels.

**Supplemental Table 1**

Demographics of contributions from each ENIGMA site.

**Supplemental Table 2**

Differences between regions of significantly reduced surface area, cortical thickness or subcortical volume in patients with schizophrenia compared to controls (FTD corrected p = 0.001).

**Supplemental Table 3.**

T Statistics of regions significantly associated with PANSS N5 (FDR corrected p = 0.001).

**Supplemental Table 4**

T statistics of regions significantly associated with total FTD, corrected for total PANSS scores (p = 0.001, FDR corrected).
