# Supplementary material for: Differences in the neural correlates of schizophrenia with positive and negative formal thought disorder in patients with schizophrenia in the ENIGMA dataset
Source: Mol Psychiatry. 2024 Apr 26;29(10):3086–96. doi: 10.1038/s41380-024-02563-z (PMC11449795; doi:10.1038/s41380-024-02563-z)
Supplement: Supplementary file 2 — Supplemental Table 1 [file 41380_2024_2563_MOESM2_ESM.docx]

| ENIGMA Site | Number of Subjects (Number of Patients) | Age | Number of Males | Average Duration of Illness | Average PANSS Total Score (StDev) | Average PANSS Positive Score (StDev) | Average PANSS Negative Score (StDev) |
| --- | --- | --- | --- | --- | --- | --- | --- |
| FIDMAG | 279 (156) | 38.85 (11.07) | 176 | 15.97 (11.23) | 75.84 (18.39) | 16.78 (5.74) | 22.67 (6.71) |
| Zurich | 37 (24) | 32.49 (9.32) | 22 | 9.51 (7.84) | 57.96 (12.05) | 16.38 (5.36) | 12.75 (4.99) |
| Marburg | 681 (40) | 32.92 (12.28) | 266 | 169.24 (119.82) | NA | NA | NA |
| Singapore | 214 (138) | 32.95 (9.38) | 145 | 6.80 (7.47) | 40.16 (8.45) | 10.70 (3.79) | 9.03 (3.12) |
| RomeSL | 280 (164) | 38.60 (11.43) | 183 | 14.91 (10.69) | NA | NA | NA |
| UCI | 145 (74) | 36.35 (11.61) | 64 | 14.60 (10.16) | 45.85 (12.29) | 10.88 (6.43) | 10.32 (4.18) |
| PHCP | 128 (46) | 43.88 (13.56) | 61 | 18.55 (11.13) | NA | NA | NA |
| PENS | 51 (17) | 47.33 (9.39) | 27 | 24.71 (10.49) | NA | NA | NA |
| GSU | 135 (65) | 36.36 (12.59) | 103 | 15.58 (12.59) | 58.71 (14.70) | 14.92 (5.24) | 14.60 (4.69) |
| UCISZ | 58 (28) | 42.11 (11.44) | 45 | 17.50 (10.05) | 59.96 (12.00) | 15.56 (4.15) | 16.04 (5.88) |
| MCIC | 88 (60) | 31.17 (8.75) | 63 | 8.35 (7.26) | 48.65 (10.41) | 10.72 (2.66) | 14.50 (5.86) |

Supplementary Table 1. Demographics of contributions from each ENIGMA site.

**Supplementary Table 1.** Demographics of contributions from each ENIGMA site.
