# Supplementary material for: Differences in the neural correlates of schizophrenia with positive and negative formal thought disorder in patients with schizophrenia in the ENIGMA dataset
Source: Mol Psychiatry. 2024 Apr 26;29(10):3086–96. doi: 10.1038/s41380-024-02563-z (PMC11449795; doi:10.1038/s41380-024-02563-z)
Supplement: Supplementary file 4 — Supplemental Table 3 [file 41380_2024_2563_MOESM4_ESM.docx]

**Supplementary Table 3.** T Statistics of regions significantly associated with PANSS N5 (FDR corrected p = 0.001).

| **Surface Area** | | | | **Cortical Thickness** | | | | **Subcortical Volume** | | | |
| --- | --- | --- | --- | --- | --- | --- | --- | --- | --- | --- | --- |
| Left Lateral Occipital | -4.52 | Right Lateral Occipital | -5.31 | Left Rostral Middle Frontal | 4.64 | Right Rostral Middle Frontal |  | Left Pallidum | -5.77 | Right Pallidum | -6.02 |
|  |  | Right Cuneus | -5.09 |  |  |  |  | Left Amygdala | -4.55 |  |  |
| Left Lateral Orbitofrontal | -4.63 |  |  |  |  |  |  |  |  |  |  |
| Left Medial Orbitofrontal | -5.12 | Right Medial Orbitofrontal | -5.68 |  |  |  |  |  |  |  |  |
|  |  | Overall Right Surface Area | -4.66 |  |  |  |  |  |  |  |  |
|  |  |  |  |  |  |  |  |  |  |  |  |
|  |  |  |  |  |  |  |  |  |  |  |  |
|  |  |  |  |  |  |  |  |  |  |  |  |
|  |  |  |  |  |  |  |  |  |  |  |  |
|  |  |  |  |  |  |  |  |  |  |  |  |
|  |  |  |  |  |  |  |  |  |  |  |  |

**Supplementary Table 3.** T Statistics of regions significantly associated with PANSS N5 (FDR corrected p = 0.001).
