# Supplementary figures and images for: Differences in the neural correlates of schizophrenia with positive and negative formal thought disorder in patients with schizophrenia in the ENIGMA dataset
Source: Mol Psychiatry. 2024 Apr 26;29(10):3086–96. doi: 10.1038/s41380-024-02563-z (PMC11449795; doi:10.1038/s41380-024-02563-z)

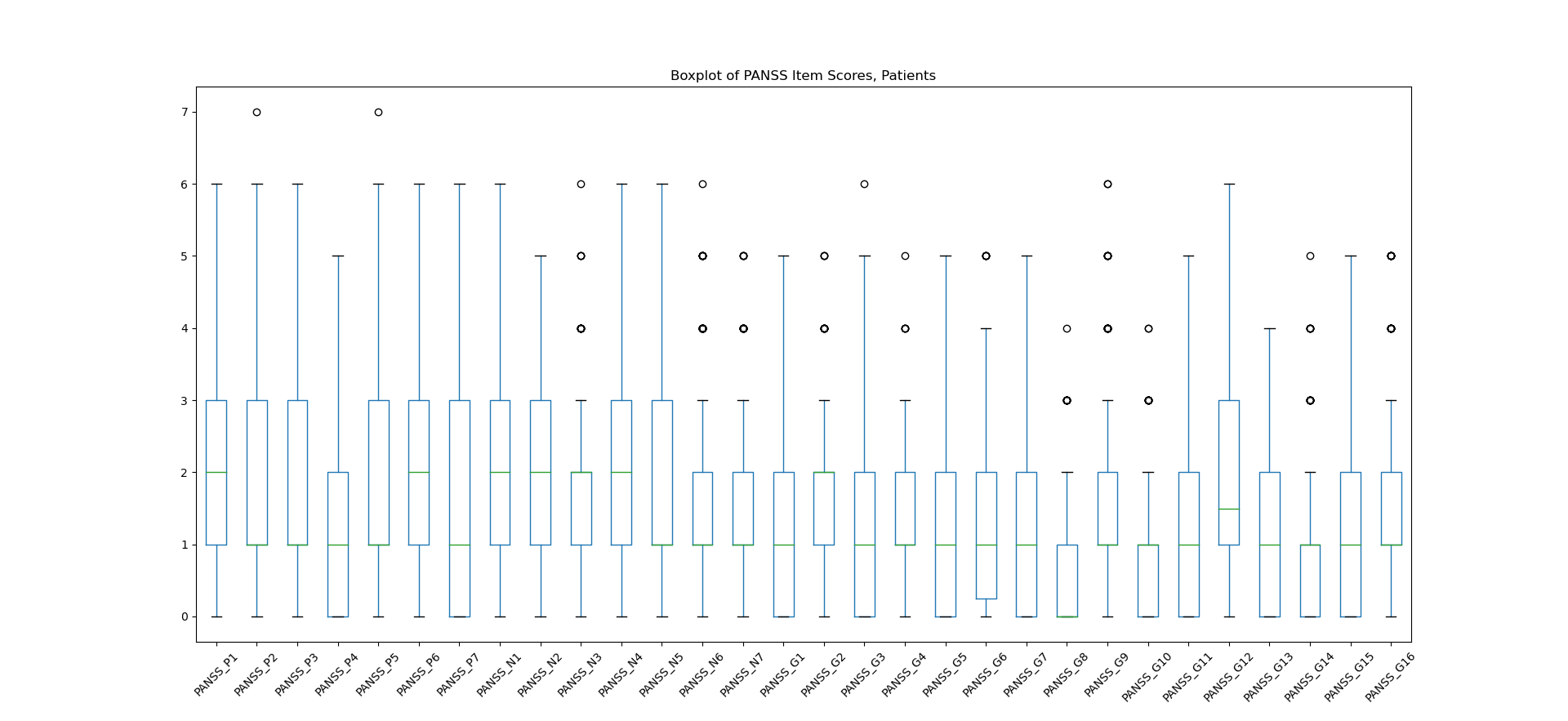

Supplement: Supplementary file 5 — Supplemental Figure 1 [file 41380_2024_2563_MOESM5_ESM.png]

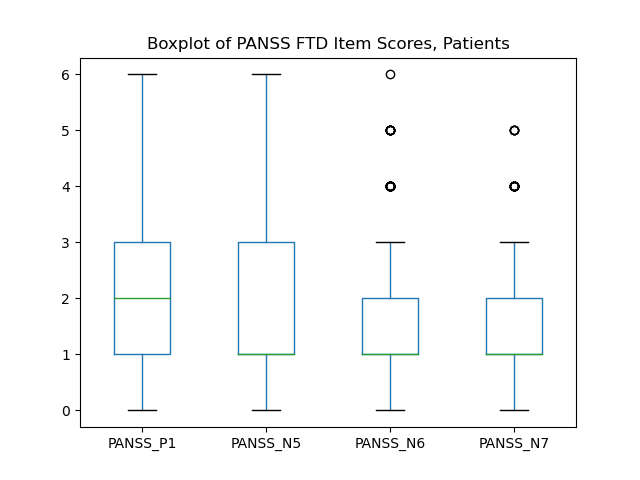

Supplement: Supplementary file 6 — Supplemental Figure 2 [file 41380_2024_2563_MOESM6_ESM.png]

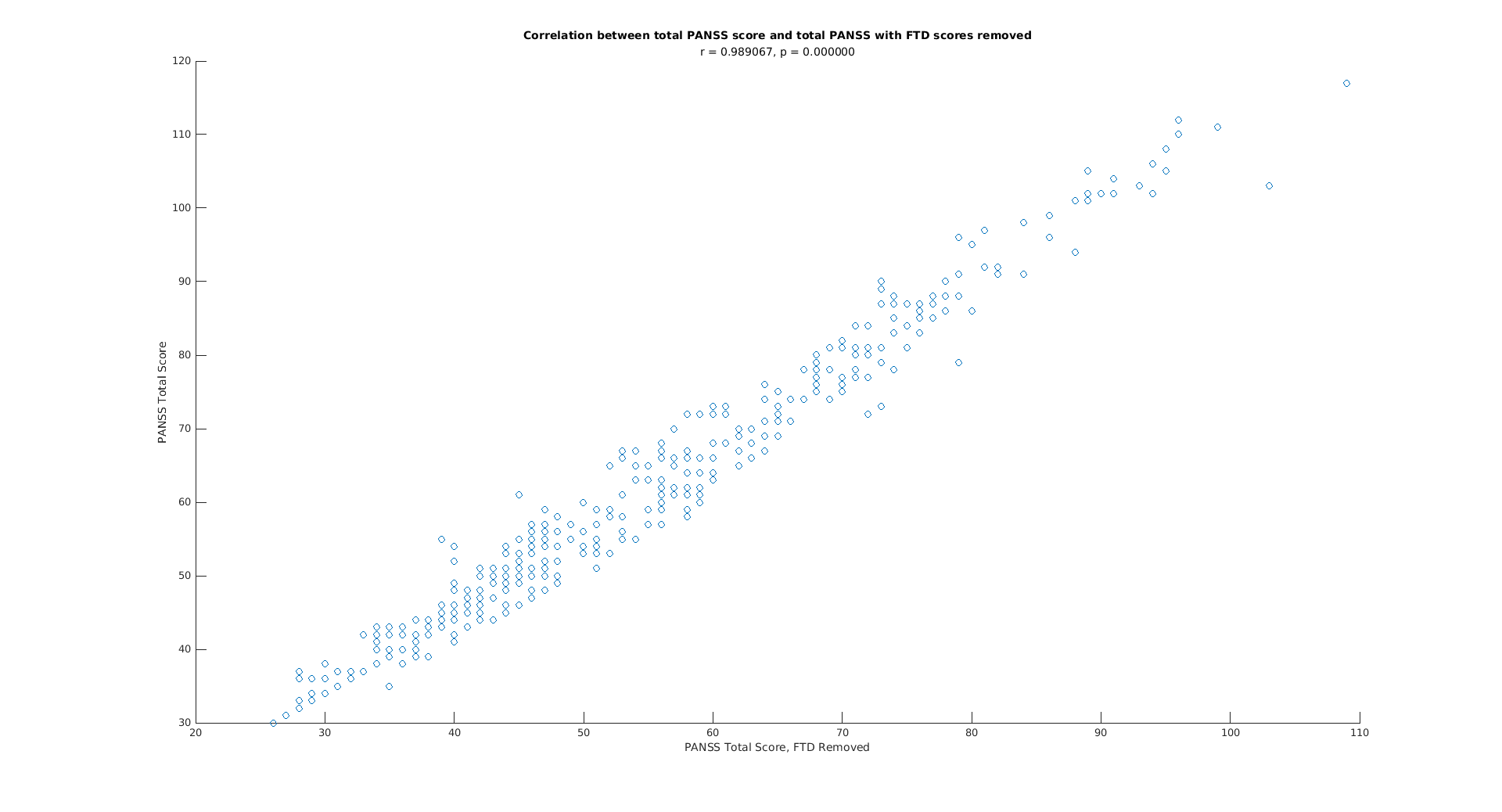

Supplement: Supplementary file 7 — Supplemental Figure 3 [file 41380_2024_2563_MOESM7_ESM.png]
